# Peri-Eyebrow Incision: A Practical and Aesthetic Solution for Forehead Lipoma Surgery

**DOI:** 10.3390/jcm14238460

**Published:** 2025-11-28

**Authors:** Dong Wan Kim, Ho Jun Lee, Seung Hyun Kim, Jun Ho Choi, Jae Ha Hwang, Kwang Seog Kim

**Affiliations:** Department of Plastic and Reconstructive Surgery, Chonnam National University Hospital, Chonnam National University Medical School, 42 Jebong-ro, Dong-gu, Gwangju 61469, Republic of Korea; waaan37@gmail.com (D.W.K.); lghk0419@naver.com (H.J.L.); hi_1004@naver.com (S.H.K.); cjh_0502@hanmail.net (J.H.C.); pskim@chonnam.ac.kr (K.S.K.)

**Keywords:** forehead, lipoma, eyebrow, surgical procedures, plastic surgery procedures

## Abstract

**Background/Objectives**: Forehead lipomas are benign but cosmetically conspicuous. Direct transcutaneous incision allows easy removal but often leaves visible scars. Hairline approaches conceal scars but are unsuitable for bald or high-hairline patients and lower forehead lipomas. We evaluated a peri-eyebrow approach using a superior brow-margin incision versus conventional methods. **Methods**: We retrospectively reviewed 176 patients who underwent forehead mass excision between 2008 and 2025. After exclusions, 97 biopsy-proven lipomas were analyzed (peri-eyebrow 22; hairline 38; direct 37). Variables included lipoma location, vertical ratio (lipoma position between the hairline and eyebrow), lipoma size, and incision length. Logistic regression assessed the relationship between vertical ratio and peri-eyebrow incision selection. Multivariate logistic regression identified independent predictors, and bootstrap validation (1000 iterations) assessed the internal stability of the ROC cut-off. Scar quality was evaluated using the Patient and Observer Scar Assessment Scales. **Results**: Peri-eyebrow incisions were used only for midline or median lipomas. The vertical ratio was highest in the peri-eyebrow group (0.70 ± 0.10) and independently predicted incision choice (adjusted OR per 0.1-unit increase 3.32, *p* < 0.001). ROC analysis showed excellent discrimination (AUC 0.872), and the bootstrapped AUC (0.802, 95% CI 0.727–0.878) confirmed robust internal validity. The optimal 0.615 cut-off yielded 0.82 sensitivity and 0.79 specificity. Peri-eyebrow and hairline incisions achieved significantly better scar scores than direct incisions (*p* < 0.001). **Conclusions**: The peri-eyebrow incision is a safe and cosmetically effective alternative when hairline incisions are unsuitable. It offers concealed scars and outcomes comparable to hairline incisions.

## 1. Introduction

Lipoma is a benign soft-tissue tumor composed of mature adipose cells enclosed in a fibrous capsule. It can develop in any region of the body and generally does not require treatment unless it enlarges rapidly, causes functional impairment, or raises suspicion of malignancy. However, lipomas located on the forehead are particularly conspicuous due to the visibility of the area, and many patients seek excision for cosmetic reasons. In such cases, achieving both complete removal and minimal scarring is of paramount importance.

Traditionally, forehead lipomas have been excised through direct transcutaneous incisions. While this approach provides straightforward access and reliable removal under direct visualization, it often results in visible scars in one of the most aesthetically sensitive facial regions [1,2]. To minimize scar visibility, alternative techniques have been developed. Hairline incisions, sometimes combined with endoscopic assistance, permit lipoma removal through concealed scalp scars and have been reported to yield excellent cosmetic outcomes [3,4,5]. Nevertheless, hairline approaches are less suitable for patients with high hairlines or baldness, or when lipomas are located in the lower forehead, where the distance from the incision to the lesion increases and dissection becomes more extensive [6].

To overcome these limitations, the peri-eyebrow incision has been proposed. This technique places the incision along the superior margin of the eyebrow, allowing the scar to be camouflaged within the brow line [2]. The approach provides a short, direct route to the lipoma—especially when the lesion lies closer to the eyebrow—while maintaining satisfactory cosmetic outcomes. Although forehead lipoma excision is a simple and commonly performed procedure, incision design in this aesthetically sensitive region has important implications for postoperative appearance and patient satisfaction. Establishing an objective and reproducible guideline for incision selection is clinically meaningful, even in this seemingly straightforward condition. To our knowledge, no peer-reviewed study has specifically evaluated peri-eyebrow incisions for forehead lipoma excision or compared this method with hairline and direct transcutaneous approaches. Therefore, the present study aimed to compare the clinical safety and cosmetic outcomes of peri-eyebrow, hairline, and direct incisions in forehead lipoma excision, and to assess the role of the vertical ratio as an objective guideline for surgical planning.

## 2. Materials and Methods

### 2.1. Subjects

A retrospective review was conducted of 176 patients who underwent forehead mass excision between January 2008 and December 2025 at Chonnam National University Hospital. After excluding non-lipoma tumors (*n* = 54), lesions located in the scalp, eyebrow, or temporal region (*n* = 15), and cases without available preoperative photographs (*n* = 10), a total of 97 patients with biopsy-confirmed forehead lipomas were included in the final analysis. Patients were categorized into three groups according to the surgical approach: peri-eyebrow incision (*n* = 22), hairline incision (*n* = 38), and direct transcutaneous incision (*n* = 37).

### 2.2. Data Collection

Clinical and demographic data were retrospectively obtained from medical records and standardized preoperative photographs. Only biopsy-confirmed forehead lipomas were included in the analysis. Surgical approaches were classified as peri-eyebrow, hairline, or direct transcutaneous incisions.

**Figure 1:**
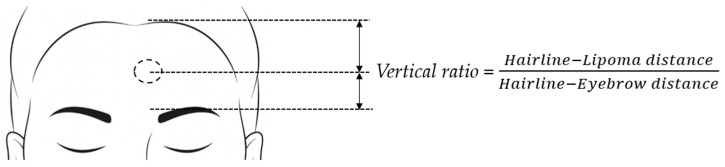
Schematic illustration of the vertical ratio, defined as the ratio of the hairline–lipoma distance to the hairline–eyebrow distance.

For each case, the vertical ratio was calculated to quantify the relative position of the lipoma between the hairline and the eyebrow. Specifically, the distance from the hairline to the lipoma and the distance from the hairline to the eyebrow were measured using standardized frontal photographs. The vertical ratio was defined as follows (Figure 1):

Because lipoma size varied among patients, all measurements were taken from the lipoma’s center point to the eyebrow and hairline to ensure consistency. This ratio normalized lipoma position across individuals with different forehead heights and provided a reproducible continuous variable for statistical analysis. To maintain measurement reliability, only frontal-view photographs with a neutral head position and clear exposure of both the hairline and eyebrow were used. Measurements were performed retrospectively using digital calipers within the hospital’s image-viewing system.

### 2.3. Study Design

**Figure 2:**
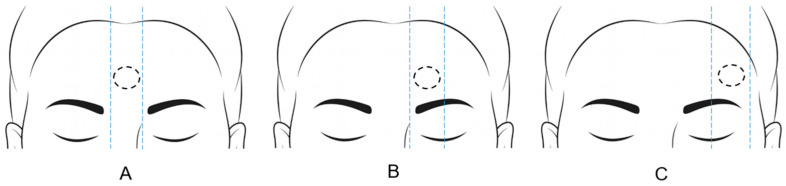
Classification of the horizontal location of forehead lipomas: (**A**) midline, when the lipoma is located across the central vertical line of the forehead; (**B**) median, when the lipoma lies between the midline and the vertical lines drawn from the midpoint of the eyebrow; (**C**) lateral, when the lipoma is positioned beyond the vertical lines drawn from the midpoint of the eyebrow toward the lateral end.

This study was designed as a retrospective, single-center cohort analysis. All cases were reviewed using medical records and standardized clinical photographs. Clinical variables, including patient age, sex, lipoma location (midline/median/lateral), vertical ratio, lipoma size, and incision length, were recorded. The horizontal location of lipomas was classified as midline, median, or lateral, as illustrated in Figure 2.

Postoperative complications such as recurrence, depression, or nerve injury were also documented. Scar outcomes were evaluated using the Patient and Observer Scar Assessment Scales (PSAS and OSAS) at 6 months postoperatively.

This study was approved by the Institutional Review Board of Chonnam National University Hospital (IRB No. CNUH-2025-324; approval date: 25 September 2025) and conducted in accordance with the principles of the Declaration of Helsinki. Written informed consent was obtained from all patients for inclusion in the study and publication of clinical data and images.

### 2.4. Operation Technique

#### 2.4.1. Direct Transcutaneous Incision

**Figure 3:**
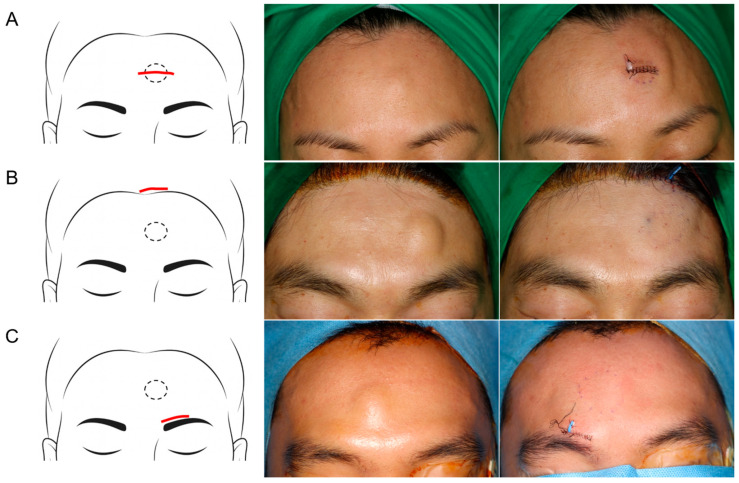
Schematic illustrations and clinical photographs of surgical approaches for forehead lipoma excision: (**A**) Direct transcutaneous incision; (**B**) Hairline incision; (**C**) Peri-eyebrow incision.

A transverse incision was designed directly over the center of the lipoma, taking into account the resting skin tension lines (RSTL). After the skin incision, the lipoma was exposed and excised under direct visualization. Layered closure was performed using 6-0 Vicryl and Dafilon sutures (Figure 3A).

#### 2.4.2. Hairline Incision

A transverse incision was made slightly posterior to the anterior hairline, closer to the scalp, to conceal the resulting scar (Figure 3B).

#### 2.4.3. Peri-Eyebrow Incision

A transverse incision was created immediately superior to the eyebrow, following the natural brow contour to minimize scar visibility (Figure 3C).

For both the hairline and peri-eyebrow incisions, after incising the skin and subcutaneous tissue, blunt dissection was performed using blunt-tipped scissors to approach the lipoma. The contralateral hand was used externally to palpate the lipoma margin, and a periosteal elevator was employed to separate first the superior surface and then the base of the lipoma from surrounding tissue. Once isolated, the lipoma was extracted using straight mosquito forceps. The cavity was irrigated, and a Penrose drain was placed. Layered closure was performed using 5-0 or 6-0 Vicryl and Dafilon sutures. A mild compression dressing was applied for approximately 2 days to prevent hematoma formation.

### 2.5. Statistical Analysis

Continuous variables were expressed as means with standard deviations and compared among the three groups using one-way analysis of variance (ANOVA) or the Kruskal–Wallis test, depending on data normality. Categorical variables were compared using the chi-square test or Fisher’s exact test. Logistic regression analysis was conducted to examine the association between vertical ratio and selection of the peri-eyebrow incision, with odds ratios (ORs) reported per 0.1-unit increase in vertical ratio along with 95% confidence intervals. Discriminative performance was assessed using receiver operating characteristic (ROC) curve analysis and the area under the curve (AUC). The optimal cut-off point was determined by maximizing Youden’s index, and sensitivity and specificity were calculated at this threshold. Scar assessment scores (PSAS and OSAS at 6 months) were compared among groups using ANOVA with post hoc testing. A *p*-value < 0.05 was considered statistically significant. All analyses were conducted using Stata/SE version 16.1 (StataCorp, College Station, TX, USA).

For additional statistical validation, multivariate logistic regression analysis was conducted to identify independent predictors associated with the selection of the peri-eyebrow incision. Variables included the vertical ratio (per 0.1 unit increase), horizontal location, lipoma size, age, and sex. To assess the internal stability of the ROC-derived cut-off, bootstrap resampling with 1000 iterations was applied. The bootstrapped AUC (95% CI) was calculated to evaluate the reproducibility of model performance.

## 3. Results

### 3.1. Patient Selection

Between January 2008 and March 2025, 176 patients underwent forehead mass excision at our institution. After excluding non-lipoma tumors (*n* = 54), lesions in the scalp, eyebrow, or temporal area (*n* = 15), and cases without available preoperative photographs (*n* = 10), a total of 97 biopsy-confirmed forehead lipomas were included in the final analysis. Patients were categorized according to the surgical approach: peri-eyebrow incision (*n* = 22), hairline incision (*n* = 38), and direct transcutaneous incision (*n* = 37).

### 3.2. Patient Characteristics

Baseline characteristics of the three groups are summarized in Table 1. There were no significant differences in age (peri-eyebrow 44.9 ± 11.6 years; hairline 45.0 ± 13.1 years; direct 43.9 ± 14.2 years, *p* = 0.982) or sex distribution (*p* = 0.984). Lipoma size was comparable among groups (long axis: 1.7–1.8 cm; short axis: 1.2–1.4 cm). However, incision length was significantly longer in the hairline group (2.57 ± 0.71 cm) compared with the peri-eyebrow (2.00 ± 0.45 cm) and direct (2.24 ± 0.96 cm) groups (*p* = 0.004).

### 3.3. Lipoma Location

The horizontal distribution of lipomas differed significantly among surgical approaches (*p* = 0.001, Fisher’s exact test) (Table 2). The peri-eyebrow approach was used exclusively for midline (22.7%) and median (77.3%) lipomas, whereas lateral lipomas were treated only with hairline (44.7%) or direct (35.1%) incisions.

### 3.4. Vertical Ratio and Incision Selection

**Figure 4:**
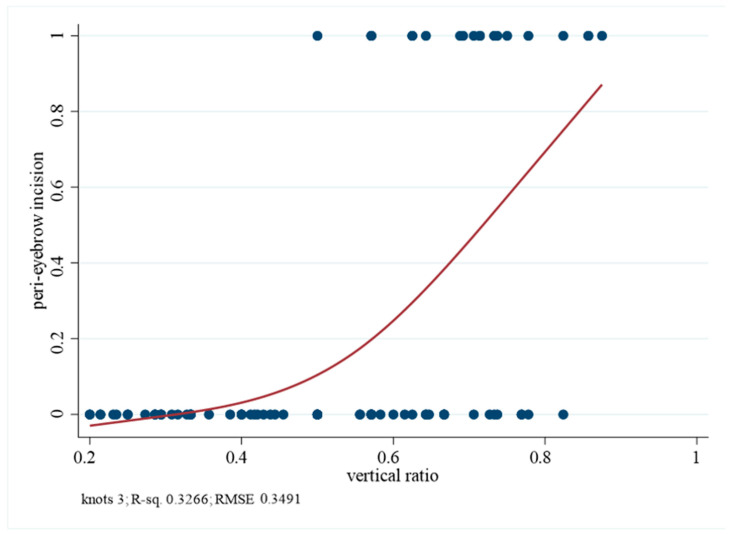
Probability of peri-eyebrow incision according to vertical ratio. Blue dots represent individual cases, and the red line indicates the logistic regression fitted curve.

**Figure 5:**
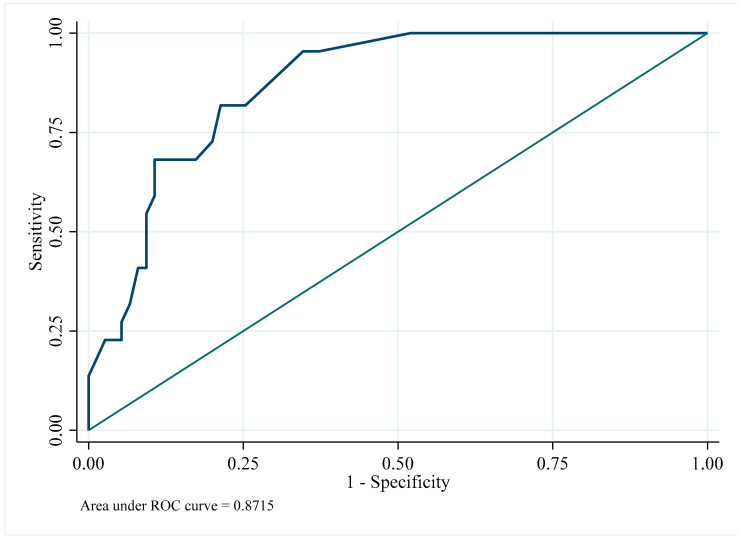
Receiver operating characteristic (ROC) curve for vertical ratio predicting peri-eyebrow incision selection (AUC = 0.872).

The vertical ratio (lipoma–eyebrow distance/hairline–eyebrow distance) was highest in the peri-eyebrow group (0.70 ± 0.10), followed by the direct (0.52 ± 0.18) and hairline groups (0.43 ± 0.14), with significant group differences (*p* < 0.001). Logistic regression analysis demonstrated that an increasing vertical ratio was strongly associated with the selection of the peri-eyebrow incision. The OR was 3.15 (95% CI 1.85–5.36, *p* < 0.001) per 0.1-unit increase in the vertical ratio. ROC curve analysis confirmed excellent discrimination (AUC = 0.872, 95% CI 0.800–0.943), and an optimal cut-off value of 0.615 yielded a sensitivity of 0.82 and specificity of 0.79. Patients with a vertical ratio greater than 0.615 were 16.6 times more likely to undergo a peri-eyebrow incision than those with a ratio ≤ 0.615 (OR 16.59, 95% CI 4.92–55.99, *p* < 0.001) (Table 3, Figure 4 and Figure 5).

### 3.5. Multivariate Analysis and Internal Validation

In the multivariate logistic regression model, the vertical ratio remained an independent predictor for selecting the peri-eyebrow incision after adjusting for horizontal location, lipoma size, age, and sex (OR = 3.32, 95% CI 1.71–6.42, *p* < 0.001) (Table 4). Internal validation of the ROC-derived cut-off value (0.615) using 1000 bootstrap resampling iterations demonstrated robust performance (AUC = 0.802, 95% CI 0.727–0.878). These results confirmed the stability and reproducibility of the vertical ratio as a reliable indicator for selecting the surgical approach.

### 3.6. Scar Outcomes

**Figure 6:**
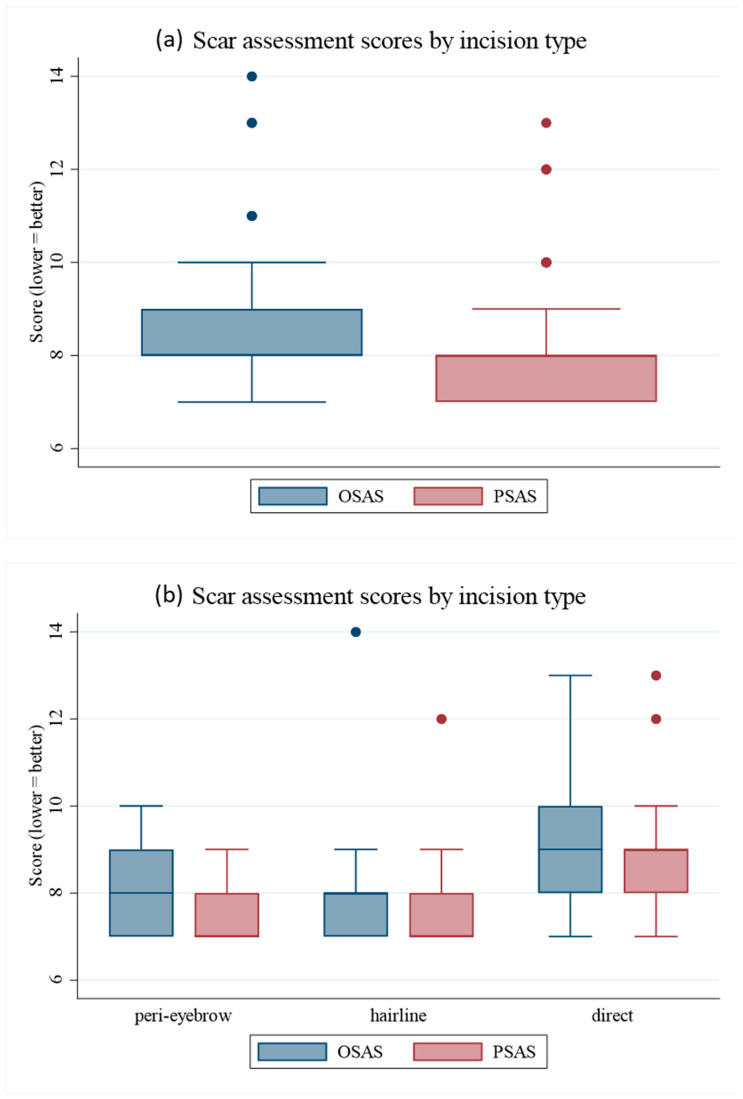
Scar assessment scores by incision type: (**a**) Distribution of OSAS and PSAS scores. (**b**) Comparison of scar assessment scores among incision types. OSAS, Observer Scar Assessment Scales; PSAS, Patient Scar Assessment Scales.

At 6 months postoperatively, scar assessments revealed significant differences among the groups (Table 5). The OSAS scores were 8.09 ± 0.87 for the peri-eyebrow group, 8.00 ± 1.21 for the hairline group, and 9.14 ± 1.46 for the direct group (*p* < 0.001). The PSAS scores were 7.45 ± 0.60, 7.50 ± 0.95, and 8.70 ± 1.27, respectively (*p* < 0.001). Post hoc analysis indicated that both peri-eyebrow and hairline incisions achieved significantly better scar scores than direct incisions, whereas no significant difference was observed between the peri-eyebrow and hairline groups. Representative boxplots of scar scores are shown in Figure 6.

### 3.7. Complications

No postoperative complications, including hematoma, infection, recurrence, or nerve injury, were observed in any group.

## 4. Discussion

### 4.1. Overall Safety and Cosmetic Outcomes

All three surgical approaches for forehead lipoma excision—direct transcutaneous, hairline, and peri-eyebrow incisions—were safe, with no postoperative complications observed [1,7]. Although the supraorbital nerve emerges from the supraorbital foramen near the eyebrow and provides sensation to the forehead and scalp, no nerve injuries occurred in this series. This finding underscores that careful incision planning and meticulous dissection along anatomical planes can preserve neurovascular structures while ensuring safe lipoma removal. Direct transcutaneous incisions, while technically straightforward, were associated with significantly inferior scar scores [1,2]. In contrast, hairline and peri-eyebrow incisions both produced favorable cosmetic outcomes, highlighting the critical role of incision design in aesthetically sensitive regions such as the forehead [3,5]. These findings are consistent with previous studies that have also emphasized the cosmetic advantages of concealed incisions along natural forehead contours and aesthetic unit boundaries [3].

### 4.2. Advantages of the Peri-Eyebrow Approach

A key finding of this study is that the peri-eyebrow incision provided scar outcomes comparable to those of the hairline approach while overcoming its inherent limitations. Hairline incisions are unsuitable for patients with baldness, high hairlines, or when lipomas are located in the lower forehead. By camouflaging the scar along the superior brow margin, the peri-eyebrow incision serves as a practical alternative in these scenarios. Logistic regression confirmed that the vertical ratio was strongly associated with peri-eyebrow selection, with the odds of choosing this approach increasing more than threefold for every 0.1-unit increment. ROC curve analysis further supported its predictive utility, yielding an AUC of 0.872 and identifying an optimal cut-point of 0.615. Clinically, this suggests that a peri-eyebrow incision should be considered when the lipoma is located at or below this relative position between the hairline and eyebrow.

### 4.3. Predictive Validation and Clinical Correlation

In this study, the peri-eyebrow incision was primarily applied to central and paramedian forehead lipomas, whereas lateral lesions were treated through hairline or direct incisions. This pattern reflects both aesthetic considerations and surgical accessibility. Multivariate analysis demonstrated that the vertical ratio remained a significant independent factor influencing incision choice, even after adjusting for horizontal location, lipoma size, and patient demographics. These findings indicate that the vertical ratio serves as a practical and reproducible parameter for preoperative planning. In all patients, preoperative ultrasonography and computed tomography (CT) were routinely performed to confirm the diagnosis and delineate the anatomical extent of the lipoma, ensuring accurate localization before incision planning [8].

Internal validation using bootstrap resampling confirmed the stability of the ROC-derived cut-off value (AUC 0.86, 95% CI 0.80–0.92). Although this strengthens the reliability of our results, the cut-off should be interpreted as exploratory because the data originated from a single institutional cohort. Further multicenter or prospective validation would help generalize these findings.

From an aesthetic perspective, both peri-eyebrow and hairline incisions achieved superior scar outcomes compared to direct transcutaneous incisions, particularly when the lesion was located near the eyebrow margin. The peri-eyebrow incision provided excellent scar concealment by following the natural brow contour, whereas the hairline incision was advantageous for higher lesions with thicker subcutaneous tissue. Overall, the vertical ratio threshold (0.615) may serve as a useful reference for choosing between these two approaches according to the lesion’s relative height, offering a simple and objective guideline for surgical decision-making.

### 4.4. Location-Specific Considerations

Notably, peri-eyebrow incisions were not performed for lateral forehead lipomas in this series [4]. This likely reflects the limited exposure achieved through a brow-margin incision in lateral regions, where hairline or direct transcutaneous approaches may be more practical. Although horizontal location was not a statistically significant predictor in multivariate analysis, the peri-eyebrow approach appeared clinically well suited for midline and median lipomas, particularly those situated closer to the eyebrow than to the hairline.

While the vertical ratio was a strong determinant of peri-eyebrow selection, some lower-forehead lipomas were still managed with direct transcutaneous incisions. These cases typically involved patients with preexisting forehead scars, very small lipomas, or lesions that were difficult to palpate, where direct visualization was preferred for precise excision. Such examples underscore that incision planning depends not only on lipoma location but also on patient-specific and intraoperative factors [7].

### 4.5. Clinical Implications and Novelty

The findings of this study align with previous reports emphasizing the cosmetic disadvantages of direct incisions and the concealment benefits of alternative approaches [1,2]. However, to our knowledge, this is the first study to propose a quantitative parameter—the vertical ratio—as a predictor for peri-eyebrow incision selection. This provides an evidence-based framework for surgical planning and expands the clinical applicability of the peri-eyebrow technique.

### 4.6. Limitations and Future Directions

This study has several limitations. Its retrospective design and single-center setting may restrict the generalizability of the findings. Although the sample size was larger than in most previous studies, it remains relatively modest. Furthermore, scar assessments at 6 months may not fully represent long-term cosmetic outcomes. The use of the Patient and Observer Scar Assessment Scales also introduces an element of subjectivity, despite efforts to standardize evaluations. Future prospective multicenter studies with extended follow-up periods are warranted to validate these results and refine the selection criteria for optimal surgical approaches.

## 5. Conclusions

The peri-eyebrow incision is a safe and cosmetically favorable alternative for forehead lipoma excision. It provides scar outcomes equivalent to the hairline approach and superior to direct transcutaneous incisions. This technique is particularly advantageous for lipomas located closer to the eyebrow than to the hairline, as indicated by higher vertical ratios, demonstrating its feasibility, reproducibility, and aesthetic value in clinical practice.

## Figures and Tables

**Table 1:** Baseline characteristics of patients undergoing forehead lipoma excision by incision type.

Variable	Peri-Eyebrow (*n* = 22)	Hairline (*n* = 38)	Direct (*n* = 37)	*p*-Value ^1^
Age (y), mean ± SD	44.9 ± 11.6	45.0 ± 13.1	43.9 ± 14.2	0.982
Sex, *n* (%)				0.984
Male	13 (59.1)	22 (57.9)	21 (56.8)	
Female	9 (40.9)	16 (42.1)	16 (43.2)	
Mass location, *n* (%)				0.001 ^2^
Midline	5 (22.7)	9 (23.7)	9 (24.3)	
Median	17 (77.3)	12 (31.6)	15 (40.5)	
Lateral	0 (0.0)	17 (44.7)	13 (35.1)	
Hairline–lipoma distance (cm), mean ± SD	5.4 ± 1.1	3.0 ± 1.3	3.9 ± 1.6	<0.001 (a > c > b)
Eyebrow-lipoma distance (cm), mean ± SD	2.3 ± 0.8	4.0 ± 1.2	3.6 ± 1.4	<0.001 (a < b, c)
Vertical ratio (lipoma–eyebrow/hairline–eyebrow), mean ± SD	0.7 ± 0.1	0.4 ± 0.1	0.5 ± 0.2	<0.001 (a > c > b)
Mass size (cm), mean ± SD				
Long axis	1.7 ± 0.5	1.8 ± 0.6	1.8 ± 0.7	0.664
Short axis	1.2 ± 0.5	1.3 ± 0.5	1.4 ± 0.7	0.496
Incision length (cm), mean ± SD	2.0 ± 0.5	2.6 ± 0.7	2.2 ± 1.0	0.004 (b > a, c)

^1^ Kruskal–Wallis rank test or Pearson chi-square test, as appropriate. ^2^ Fisher’s exact test.

**Table 2:** Horizontal distribution of forehead lipomas according to incision type.

Location	Peri-Eyebrow (*n* = 22)	Hairline (*n* = 38)	Direct (*n* = 37)	*p*-Value
Midline	5 (22.7%)	9 (23.7%)	9 (24.3%)	
Median	17 (77.3%)	12 (31.6%)	15 (40.5%)	
Lateral	0 (0.0%)	17 (44.7%)	13 (35.1%)	<0.001 ^1^

^1^ Fisher’s exact test. The peri-eyebrow approach was applied exclusively to midline and median lipomas, whereas lateral lipomas were treated only with hairline or direct incisions (*p* = 0.001).

**Table 3:** Logistic regression and receiver operating characteristic (ROC) curve analysis for vertical ratio predicting peri-eyebrow incision.

Analysis	Odds Ratio (95% CI)	*p*-Value ^1^
Logistic regression (per 0.1-unit increase)	3.15 (1.85–5.36)	<0.001
Logistic regression (cut-off 0.615, categorical)	16.59 (4.92–55.99)	<0.001

^1^ Logistic regression analysis. ROC curve analysis. Area under the curve (AUC) = 0.872 (95% CI 0.800–0.943). Optimal cut-off = 0.615 (Liu’s method). Sensitivity = 0.82. Specificity = 0.79. Values are presented as odds ratios (95% CI). Higher vertical ratios were strongly associated with peri-eyebrow incision (odds ratio per 0.1-unit increase 3.15, *p* < 0.001). ROC analysis demonstrated excellent discrimination (AUC = 0.872), with a cut-off value of 0.615 yielding sensitivity of 0.82 and specificity of 0.79.

**Table 4:** Multivariate logistic regression and bootstrap validation for predicting peri-eyebrow incision selection.

Analysis	Variable	OR (95% CI)	*p*-Value
Multivariate logistic regression ^1^	Vertical ratio (per 0.1-unit increase)	3.32 (1.71–6.42)	<0.001
	Mass location—median (vs. midline)	3.91 (0.75–20.40)	0.105
	Mass size (long axis, cm)	0.59 (0.19–1.78)	0.349
	Age (y)	1.00 (0.94–1.06)	0.989
	Sex—female (vs. male)	0.44 (0.11–1.81)	0.259
Bootstrap validation (1000 iterations) ^2^	Cut-off value	0.62 (0.53–0.71)	-
	AUC	0.80 (0.73–0.88)	-

^1^ Multivariate logistic regression analysis was performed to identify independent predictors associated with the selection of the peri-eyebrow incision. ^2^ Bootstrap resampling with 1000 iterations was conducted to assess the internal stability of the ROC-derived cut-off value and model discrimination performance.

**Table 5:** Scar assessment scores (OSAS and PSAS) by incision type at 6 months postoperatively.

	Peri-Eyebrow (*n* = 22)	Hairline (*n* = 38)	Direct (*n* = 37)	*p*-Value ^1^
OSAS	8.1 ± 0.9	8.0 ± 1.2	9.1 ± 1.5	<0.001
PSAS	7.5 ± 0.6	7.5 ± 1.0	8.7 ± 1.3	<0.001

^1^ Kruskal–Wallis rank test with Bonferroni post hoc correction. Values are presented as mean ± SD. Direct incisions had significantly worse OSAS and PSAS scores compared with peri-eyebrow and hairline approaches (*p* < 0.001). No significant difference was found between peri-eyebrow and hairline groups. OSAS, Observer Scar Assessment Scales; PSAS, Patient Scar Assessment Scales.

## Data Availability

The data presented in this study are available on request from the corresponding author. The data are not publicly available due to privacy or ethical restrictions.

## Abbreviations

The following abbreviations are used in this manuscript:

ANOVA	Analysis of variance
AUC	Area under the curve
CT	Computed tomography
ORs	Odd ratios
OSAS	Observer Scar Assessment Scales
PSAS	Patient Scar Assessment Scales
ROC	Receiver operating characteristic
RSTL	Resting skin tension lines
